# Association between serum HMGB1 elevation and early pediatric acute respiratory distress syndrome: a retrospective study of pediatric living donor liver transplant recipients with biliary atresia in China

**DOI:** 10.1186/s12871-023-02040-0

**Published:** 2023-03-21

**Authors:** Yimei Cao, Jiahao Zhi, Hengchang Ren, Mingwei Sheng, Lili Jia, Yiqi Weng, Hongyin Du, Wenli Yu

**Affiliations:** 1 The First Central Clinical College of Tianjin Medical University, Tianjin, 300070 China; 2 Department of Anesthesiology, Tianjin First Central Hospital, 24 Fukang Road, Nankai District, Tianjin, 300192 China

**Keywords:** HMGB1, PARDS, Liver transplantation, Biliary atresia

## Abstract

**Background:**

High mobility group box 1 (HMGB1) protein is one of the main risk factors for pediatric acute respiratory distress syndrome (PARDS) after living donor liver transplantation (LDLT). However, studies of the relationship between HMGB1 and PARDS are lacking. We evaluated the link between anomalies of intraoperative serum HMGB1 and PARDS in pediatric LDLT recipients with biliary atresia during the first week after transplant.

**Methods:**

Data for 210 pediatric patients with biliary atresia who underwent LDLT between January 2018 and December 2021 were reviewed retrospectively. The main measure was serum HMGB1 levels 30 min after reperfusion, while the outcome was early PARDS after LDLT. Data including pretransplant conditions, laboratory indexes, variables of intraoperation, clinical complications, and outcomes after LDLT were analyzed for each patient. Univariate analysis of PARDS and multivariate logistic regression analyses of serum HMGB1 levels at 30 min in the neohepatic phase in the presence of PARDS were conducted to examine the potential associations. Subgroup interaction analyses and linear relationships between intraoperative serum HMGB1 levels and PARDS were also performed.

**Results:**

Among the participants, 55 had PARDS during 7 days after LDLT, including four in the first HMGB1 tertile (4.3–8.1 pg/mL), 18 in the second tertile (8.2–10.6 pg/mL), and 33 in the third tertile (10.6–18.8 pg/mL). The nonadjusted association between intraoperative HMGB1 levels and PARDS was positive (odds ratio 1.41, 95% confidence intervals 1.24–1.61, *P* < 0.0001). The association remained unchanged after adjustment for age, weight, pretransplant total bilirubin, albumin, graft cold ischemia time, and intraoperative blood loss volume (odds ratio 1.28, 95% confidence interval 1.10–1.49, *P* = 0.0017). After controlling for potential confounders, the association between intraoperative HMGB1 levels and PARDS remained positive, as well as in the subgroup analyses.

**Conclusions:**

Serum HMGB1 levels at 30 min after reperfusion were positively associated with early PARDS among pediatric patients with biliary atresia who had undergone LDLT. Identifying such patients early may increase the efficacy of perioperative respiratory management.

**Supplementary Information:**

The online version contains supplementary material available at 10.1186/s12871-023-02040-0.

## Background

Biliary atresia is a serious congenital anomaly characterized by persistent and progressive cholestatic jaundice and if left untreated can result in end-stage liver failure that is life-threatening [1]. The incidence of biliary atresia varies greatly between the Eastern and Western world. It is more common in East Asia, especially China, with an incidence of 2 per 10,000 live births [2]. Liver transplantation is the only effective treatment for pediatric biliary atresia [3, 4]. With improvements in surgery and anesthesia management techniques, the 10-year survival rate of pediatric liver transplantation has reached more than 85% [5, 6]. Most donors are parents for living donor liver transplantation (LDLT). However, distant organ damage, affecting the heart, brain, kidneys, lungs, and intestines, is still an important factor affecting the long-term survival of children after surgery [7, 8].

The lung is especially susceptible to proinflammatory mediators released by the injured liver during the period of perioperative liver transplantation [9]. Pediatric acute respiratory distress syndrome (PARDS) was defined in children in 2015 as the acute onset of parenchymal lung disease on a chest x-ray with severe hypoxemia but not explained by cardiac disease [10]. It is a common complication after liver transplantation. Perioperative pulmonary complications are the main cause of perioperative mortality and prolonged hospital stay in pediatric liver transplantation recipients, with an incidence of more than 20% [11, 12].

High mobility group box 1 (HMGB1) protein is a mediator of inflammation. HMGB1 is referred to as a damage-associated molecular pattern, which is a general term for endogenous danger signals released by the body after injury. Liver ischemia-reperfusion injury triggers HMGB1 release [13]. In addition, extensive studies have demonstrated that plasma HMGB1 level is a sensitive indicator of acute respiratory distress syndrome (ARDS) [14]. HMGB1 is a central mediator of lethal inflammation and could be a potential target for innovative therapeutic strategies for COVID-19 [15, 16], even in the pediatric field [17].

Considerable progress has been made in the diagnosis and treatment of PARDS after liver transplantation in recent years, but challenges remain. One challenge is predicting the prognosis and providing targeted therapies for PARDS in LDLT recipients with biliary atresia. In addition, limited research has demonstrated the relationship between HMGB1 and PARDS after LDLT in children with biliary atresia.

In this study, we conducted a detailed evaluation of the association between intraoperative HMGB1 elevation and PARDS during the first week after LDLT in children with biliary atresia in China.

## Methods

### Participants

This retrospective cohort study adhered to the STROBE reporting guidelines [18]. The study was approved by the ethics committee of Tianjin First Central Hospital in China (Approval Number: 2020N261KY). The written informed consent was waived by Tianjin First Central Hospital Ethics Committee owing to the retrospective design. We reviewed the medical records of all pediatric liver transplantation recipients from January 2018 to December 2021 in Tianjin First Central Hospital in China. The operation was performed by experienced surgeons and the procedure was a piggyback liver transplant.

Inclusion criteria were as follows: (I) the diagnosis was biliary atresia; (II) living donors were either the father or the mother; and (III) the level of serum HMGB1 had been obtained. Exclusion criteria were as follows: (I) the preoperative diagnosis was not biliary atresia; (II) the donor was not a parent; (III) the patient had perinatal disease, congenital cardiopulmonary deformity, or acute respiratory infection before surgery; (IV) the patient had hepatopulmonary syndrome; (V) retransplantation; and (VI) the serum HMGB1 measurements were unavailable.

### Data collection

Perioperative data were obtained from an institutionally maintained archive of perioperative data and electronic medical records. Supplemental electronic searches or manual examinations were completed if needed.

Demographic data, pretransplant conditions, laboratory indices, intraoperative variables, clinical complications, and outcomes after LDLT were collected. In addition, donor information was also collected.

The clinical and laboratory data were collected by a dedicated research nurse as part of the standard of care in our hospital. In this study, the concentrations of serum HMGB1 were analyzed by enzyme-linked immunosorbent assay (reagent kit provided by Shanghai Biovol Biotechnology Co., Shanghai, China). A sample is repeated three times and then averaged to ensure the accuracy and precision of the measurement results. In addition, all tests are conducted in the same batch of reagent kits in the same hospital.

### The definition of PARDS after pediatric liver transplantation

We used the definition of PARDS recommended by the 2015 Pediatric Acute Lung Injury Consensus Conference developed at an Expert Consensus Meeting [10]. Specifically, the diagnostic criteria for PARDS are shown in Table 1. In pediatric patients, arterial partial pressure of oxygen (PaO_2_)-based metrics are preferred if available. If PaO_2_ is not available, the fraction of inspiration O_2_ (FiO_2_) is weaned to maintain pulse oxygen saturation (SpO_2_) 97% to calculate the oxygen saturation index (OSI) or the SpO_2_/FiO_2_ ratio.

**Table 1:** Details of PARDS [10].

Name	Description		
**Age**	Exclude patients with perinatal-related lung disease		
**Timing**	Within 7 days of known clinical insult;		
**Origin of edema**	Respiratory failure not fully explained by cardiac failure or fluid overload		
**Chest imaging**	Chest imaging findings of new infiltrate(s) consistent with acute pulmonary parenchymal disease;		
**Oxygenation**	**Noninvasive mechanical ventilation**		
	PARDS (No severity stratification)		
	Full face-mask bi-level ventilation or CPAP ≥ 5 cm H_2_O PF ratio ≤ 300 or SP ratio ≤ 264		
	**Invasive mechanical ventilation**		
	Mild	Moderate	Severe
	4 ≤ OI < 8 or 5 ≤ OSI < 7.5	8 ≤ OI < 16 or 7.5 ≤ OSI < 12.3	OI ≥ 16 or OSI ≥ 12.3

Abbreviations: PARDS, pediatric acute respiratory distress syndrome; CPAP, continuous positive airway pressure; PF ratio, partial pressure of oxygen (PaO_2_)/fraction of inspiration O_2_ (FiO_2_); SF ratio, pulse oxygen saturation (SpO_2_)/FiO_2_; OI, oxygenation index; OSI, oxygen saturation index

OI = (FiO_2_ × mean airway pressure × 100)/PaO_2_. OSI = (FiO_2_ × mean airway pressure × 100)/SpO_2_

### Statistical analysis

The information for each patient was anonymized and de-identified before analysis. As to our knowledge, no previous studies have evaluated the association between serum HMGB1 at 30 min after reperfusion during pediatric liver transplantation and postoperative PARDS within 7 days. As a result, a formal sample size calculation was deemed not possible. According to previous publications [19], we estimated the incidence of postoperative PARDS within 7 days was 21% while HMGB1 levels were at the mean level. A logistic regression of a binary response variable (PARDS or non-PARDS) on a continuous variable (serum HMGB1 levels at 30 min after reperfusion) with a sample size of 207 observations achieves 80% power at a 0.05 significance level to detect a change in the present of PARDS from the value of 0.21 at the mean of serum HMGB1 level at 30 min after reperfusion to 0.30 when serum HMGB1 level was increased to one standard deviation above the mean [20]. Finally, 210 children with biliary atresia were analyzed in our study. Sample size calculation was performed using logistic regression with PASS 15.0 (NCSS, LLC, Kaysville, UT) software. Continuous variables are presented in the form of mean ± standard deviation for normal distribution or as median with interquartile ranges for skewed distribution, while categorical variables are presented as percentages.

The baseline characteristics of pediatric participants were categorized according to intraoperative serum levels of HMGB1 (1st tertile 4.3–8.1 pg/mL; 2nd tertile 8.2–10.6 pg/mL; and 3rd tertile 10.6–18.8 pg/mL) to detect the associated factors. For categorical variables, the analysis of variance and Chi-square tests were conducted to analyze differences between the tertiles. Afterwards, univariate analysis was performed to evaluate the associations between intraoperative HMGB1 and PARDS by using independent t-tests and Chi-square tests for continuous variables and categorical variables.

To better understand the association between intraoperative HMGB1 levels and PARDS, we conducted a multivariate logistic regression analysis of HMGB1 levels at 30 min in the neohepatic phase and postoperative PARDS by constructing three models: non-adjusted, adjust I model (variables: age and weight), and adjust II model (variables: age, weight, pretransplant albumin, total bilirubin, graft cold ischemia time, and intraoperative blood loss volume). Our covariates were selected a priori based on our previous work and studies from other researchers examining risk factors for PARDS after liver transplantation. Finally, the dependent variables in univariate analysis with *P* < 0.08 were put into the multiple regression model as confounding factors for adjustment. Then, we further performed an analysis of the effect size of intraoperative HMGB1 in subgroups including age, weight, pretransplant albumin, total bilirubin, graft cold ischemia time, and intraoperative blood loss volume. Interactions were tested with correction for confounding factors. A spine plot of the probable association between intraoperative HMGB1 levels and postoperative PARDS was drawn using a generalized additive model, adjusted for weight, age, pretransplant albumin, total bilirubin, graft cold ischemia time, and intraoperative blood loss volume. *P* values equal to or less than 0.05 (two-sided) were considered statistically significant in all analyses. All of the analyses were conducted using R software version 3.4.3 (The R Foundation for Statistical Computing, Auckland, New Zealand). Several R packages (mgcv, ordinal, ggplot, stringr, multcomp, ggbeeswarm, and ggpubr) were used in the analyses.

## Results

### Pretransplant baseline characteristics of participants

**Fig. 1:**
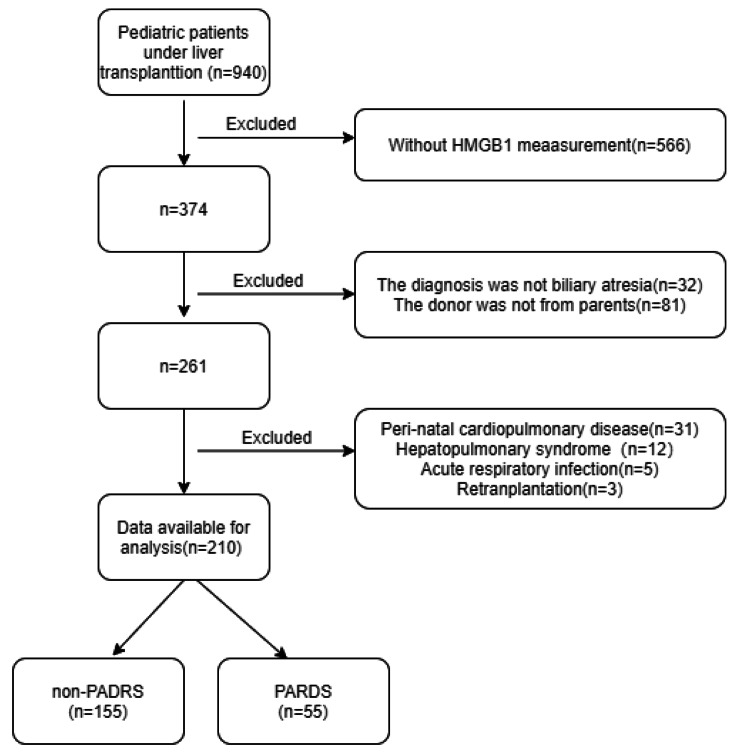
Flowchart of pediatric patients

From January 2018 to December 2021, 940 patients underwent liver transplantation. Among the 940 patients, 374 children with HMGB1 measurement were in our initial cohort. Of these, 32 were excluded because the diagnosis was not biliary atresia, 81 were excluded because the donor was not a parent, 31 were excluded because of perinatal cardiopulmonary disease, 12 were excluded because of hepatopulmonary syndrome, and five and three were excluded because of acute respiratory infection and retransplantation, respectively. Of the 210 (55.23% boys) pediatric patients who met the inclusion criteria, 55 developed PARDS during 7 days after LDLT. These comprised 38 patients with mild PARDS, 11 with moderate PARDS, three with severe PARDS, and two with no severity stratification (Fig. 1). The occurrence of early PARDS after LDLT was 26.19%.

Table 2 shows comparison of the baseline characteristics of pediatric patients by intraoperative serum HMGB1 tertiles. The difference among intraoperative HMGB1 levels tertiles was statistically significant (*P* < 0.001). Moreover, children in the 3rd tertile with elevated intraoperative HMGB1 levels showed the highest level of serum total bilirubin in the three groups before LDLT (*P* = 0.002). During transplantation, graft cold ischemia time (*P* = 0.015), surgery time (*P* = 0.015), blood loss volume (*P* < 0.001), fresh plasma transfusions (*P* < 0.001), and RBC transfusions (*P* < 0.001) were also statistically significant among different HMGB1 tertiles.

**Table 2:** Baseline characteristics of the participants by intraoperative serum high mobility group box 1 tertiles

Characteristics	Serum HMGB1, pg/mL			*p*
	1st tertile (4.3–8.1 pg/mL) (n = 70)	2nd tertile (8.2–10.6 pg/mL) (n = 69)	3rd tertile (10.6–18.8 pg/mL) (n = 71)	
Recipient				
Intraoperative HMGB1: Median (IQR), pg/mL	7.3 (6.5 to 7.7)	9.3 (8.6 to 9.8)	12.6 (11.9 to 14.0)	< 0.001
Sex, n (%)				
Male	39 (55.7)	41 (59.4)	36 (50.7)	0.581
Female	31 (44.3)	28 (40.6)	35 (49.3)	
Age: Median (IQR), month	7.8 (6.1 to 12.4)	7.6 (6.0 to 9.9)	6.5 (5.8 to 8.6)	0.102
Weight: Median (IQR), kg	7.0 (6.0 to 8.9)	7.0 (6.3 to 8.0)	6.7 (6.0 to 7.7)	0.637
Pretransplant PELD scores: Median (IQR)	16.0 (8.3 to 22.0)	17.0 (11.0 to 24.0)	21.0 (13.5 to 25.5)	0.061
Pretransplant hemoglobin: Mean ± SD, g/L	96.2 ± 12.4	95.3 ± 15.8	94.9 ± 15.2	0.858
Pretransplant creatinine: Median (IQR), µmol/L	14.0 (12.0 to 16.0)	13.0 (11.0 to 16.0)	13.0 (11.8 to 17.0)	0.398
Pretransplant albumin: Median (IQR), g/L	34.7 (32.6 to 37.6)	34.5 (31.7 to 38.6)	33.70 (31.4 to 36.2)	0.231
Pretransplant total bilirubin: Median (IQR), µmol/L	209.99 (80.6 to 324.1)	261.82 (157.8 to 337.9)	315.32 (203.4 to 487.6)	0.002
Pretransplant INR: Median (IQR)	1.4 (1.1 to 1.7)	1.4 (1.1 to 1.8)	1.4 (1.2 to 1.9)	0.251
Pretransplant WBC: Median (IQR), ×10^9^	10.6 (7.7 to 14.4)	12.0 (8.9 to 16.2)	12.7 (9.0 to 18.5)	0.244
Pretransplant lactic acid: Median (IQR), mmol/L	3.4 (2.8 to 4.5)	3.4 (2.7 to 4.2)	3.5 (2.9 to 4.6)	0.776
Pretransplant blood ammonia: Median (IQR), µmol/L	63.5 (51.0 to 84.5)	60.00 (47.0 to 79.0)	56.0 (48.0 to 76.0)	0.264
Surgery history, n (%)				0.911
No	3 (4.3)	2 (2.9)	4 (5.6)	
Yes	67(95.7)	67(97.1)	67(94.4)	
Pretransplant HMGB1: Median (IQR), pg/mL	0.9 (0.5 to 1.3)	0.9 (0.7 to 1.3)	0.8 (0.5 to 1.2)	0.472
Graft cold ischemia time: Median (IQR), min	86.5 (71.0 to 99.0)	83.0 (66.0 to 104.0)	93.0 (77.5 to 121.5)	0.015
Intraoperative blood loss volume: Median (IQR), mL	300.0 (200.0 to 400.0)	300.0 (200.0 to 400.0)	400.0 (300.0 to 600.0)	< 0.001
Intraoperative anthepatic phase time: Median (IQR), min	45.0 (39.0 to 57.0)	41.0 (38.0 to 51.0)	44.0 (37.0 to 54.0)	0.245
Intraoperative freshplasma transfusions: Median (IQR), mL	150.0 (0.0 to 400.0)	200.0 (130.0 to 400.0)	360.0 (200.0 to 400.0)	< 0.001
Intraoperative RBC transfusions: Median (IQR), in unit	2.0 (2.0 to 2.0)	2.0 (2.0 to 3.0)	2.0 (2.0 to 4.0)	< 0.001
Intraoperative urine volume: Median (IQR), mL	400.0 (300.0 to 600.0)	400.0 (300.0 to 600.0)	400.0 (240.0 to 600.0)	0.853
Intraoperative infusion volume: Median (IQR), mL	1307.5 (900.0 to 1669.0)	1130.0 (830.0 to 1466.0)	1117.5 (815.0 to 1434.0)	0.101
Intraoperative surgery time: Median (IQR), min	512.5 (455.0 to 553.0)	475.0 (414.0 to 532.0)	460.0 (412.5 to 527.5)	0.015
Donor				
Age: Median (IQR), years	31.6 (28.8 to 34.9)	30.0 (26.5 to 35.2)	30.0 (26.8 to 33.5)	0.375
Sex, n (%)				0.309
Male Female	38 (54.3) 32 (45.7)	44 (63.8) 25 (36.2)	47 (66.2) 24 (33.8)	
BMI: Median (IQR)	23.1 (21.2 to 24.8)	22.9 (20.0 to 24.9)	21.6 (19.6 to 24.7)	0.221
Weight of graft: Median (IQR), g	239.0 (212.0 to 272.0)	240.0 (213.0 to 270.0)	230.0 (200.0 to 263.5)	0.510
GRWR: Mean ± SD, %	3.4 ± 1.0	3.4 ± 0.8	3.4 ± 0.8	0.975

An analysis of variance (for continuous variables) and the chi-square test (for categorical variables) were performed to identify differences among tertiles

Abbreviations: HMGB1, high mobility group box 1; IQR, interquartile range; PELD, pediatric end-stage liver disease; INR, international normalized ratio; WBC, white blood cell; RBC, red blood cell; BMI, body mass index; GRWR: graft to recipient weight ratio

### Postoperative clinical complications and outcomes

The clinical complications and outcomes after LDLT are shown in Table 3. During the 7 days after transplantation, the rate of PARDS was 5.7%, 26.1%, and 46.5% in the 1st, 2nd, and 3rd tertile groups of increasing HMGB1 levels at 30 min in the neohepatic phase, respectively. The differences between these groups were significant (*P* < 0.001). Correspondingly, there were significant differences in postoperative ICU stay time and postoperative respiratory support time among the three groups (*P* < 0.001). However, the remaining factors of postoperative complications and outcomes were not significantly different among intraoperative HMGB1 tertiles (*P* > 0.05).

**Table 3:** Clinical complications and outcomes after pediatric liver transplantation

Outcomes	Serum HMGB1, ng/mL			P
	1st tertile (4.3–8.1 pg/mL) (n = 70)	2nd tertile(8.2–10.6 pg/mL) (n = 69)	3rd tertile (10.6–18.8 pg/mL) (n = 71)	
PARDS: n (%)				< 0.001
No	66 (94.3)	51 (73.9)	38 (53.5)	
Yes	4 (5.7)	18 (26.1)	33 (46.5)	
Mortality at 30 days after surgery: n (%)				0.276
No	70 (100)	68 (98.6)	68 (95.8)	
Yes	0 (0)	1 (1.4)	3 (4.2)	
Biliary/intestinal fistula: n (%)				0.666
No	66 (94.3)	65 (94.2)	69 (97.2)	
Yes	4 (5.7)	4 (5.8)	2 (2.8)	
Ileus: n (%)				0.762
No	66 (94.3)	63 (91.3)	65 (91.5)	
Yes	4 (5.7)	6 (8.7)	6 (8.5)	
Rejection reaction: n (%)				0.249
No	59 (84.3)	52 (75.4)	52 (73.2)	
Yes	11 (15.7)	17 (24.6)	19 (26.8)	
Lymphatic leakage: n (%)				0.140
No	41 (58.6)	50 (72.5)	51 (71.8)	
Yes	29 (41.4)	19 (27.5)	20 (28.2)	
Postoperative hospital stay time: Median (IQR), d	19.0 (14.0 to 24.0)	18.0 (14.0 to 25.0)	21.0 (15.5 to 26.0)	0.127
Postoperative ICU stay time: Median (IQR), d	3.0 (2.0 to 3.0)	2.0 (2.0 to 3.0)	3.0 (2.0 to 4.0)	< 0.001
Postoperative respiratory support time: Median (IQR), min	179.0 (143.0 to 305.0)	195.0 (154.0 to 365.0)	270.0 (175.5 to 637.5)	< 0.001

An analysis of variance (for continuous variables) and the chi-square test (for categorical variables) were performed to identify differences among tertiles

Abbreviations: HMGB1, high mobility group box 1; PARDS, pediatric acute respiratory distress syndrome; IQR, interquartile range

### Univariate analysis of PARDS

Results of the univariate analysis of PARDS during 7 days after transplantation are shown in Table 4. We found significant differences between participants with and without PARDS in terms of intraoperative HMGB1 levels, intraoperative blood loss volume, pretransplant total bilirubin, and graft cold ischemia time. Compared with the non-PARDS group, the PARDS group had significantly higher intraoperative HMGB1 levels (as a continuous variable 11.6 ± 2.5 pg/mL vs. 9.1 ± 2.5 pg/mL; *P* < 0.001). A similar trend was found for intraoperative blood loss volume (*P* = 0.001), pretransplant total bilirubin (*P* < 0.001), and graft cold ischemia time (*P* = 0.047).

**Table 4:** Univariate analysis of pediatric acute respiratory distress syndrome

Characteristic	PARDS	Non-PARDS	*P*
Weight(kg)	7.3 (2.2)	7.5 (2.1)	0.075
Age(month)	9.1 (6.3)	9.9 (6.3)	0.038
Sex: n (%)			0.453
Male	29 (25.0)	87 (75.0)	
Female	26 (27.7)	68 (72.3)	
Intraoperative HMGB1(pg/mL)	11.6 (2.5)	9.1 (2.5)	< 0.001
Intraoperative HMGB1 in tertile(pg/mL)			
1st	4 (5.7)	66 (94.3)	
2nd	18 (26.1)	51 (73.9)	
3rd	33 (46.5)	38 (53.5)	
Intraoperative blood loss volume, (mL)	415.8 (176.4)	325.0 (144.8)	0.001
Pretransplant PELD score	20.3 (9.8)	17.2 (11.2)	0.115
Pretransplant hemoglobin (g/L)	94.5 (15.0)	95.8 (14.3)	0.635
Pretransplant creatinine(µmol/L)	14.2 (4.5)	14.6 (6.3)	0.681
Pretransplant albumin(g/L)	33.7 (4.0)	35.3 (5.0)	0.052
Pretransplant total bilirubin(µmol/L)	357.2 (192.8)	233.8 (150.7)	< 0.001
Pretransplant INR	1.6 (0.6)	1.6 (0.9)	0.468
Pretransplant HMGB1: (pg/mL)	0.9 (0.5)	1.0 (0.5)	0.453
Intraoperative anthepatic phase time(min)	47.5 (13.8)	46.8 (13.1)	0.685
Intraoperative fresh plasma transfusions(mL)	251.4 (194.2)	238.1 (183.5)	0.429
Surgery history: n (%)			0.315
No	1(11.1)	8(88.9)	
Yes	54(26.9)	147(73.1)	
Intraoperative RBC transfusions (in unit)	2.6 (1.2)	2.4 (1.0)	0.087
Intraoperative urine volume(mL)	459.3 (298.5)	472.8 (319.8)	0.853
Intraoperative infusion volume(mL)	1223.0 (525.6)	1217.8 (468.4)	0.442
Intraoperative surgery time(min)	503.0 (133.5)	484.1 (87.2)	0.263
Pretransplant WBC (×10^9^)	13.8 (5.9)	13.0 (7.5)	0.232
Graft cold ischemia time(min)	104.1 (34.9)	91.3 (39.6)	0.047
Pretransplant lactic acid(mmol/L)	3.7 (1.1)	3.7 (1.3)	0.863
Pretransplant blood ammonia(µmol/L)	65.9 (26.4)	65.0 (25.1)	0.772

Analyses of the independent t test (for continuous variables) and the chi-square test (for categorical variables) were performed for univariate analysis. The variables are presented as the mean (standard deviation)

Abbreviations: PARDS, pediatric acute respiratory distress syndrome; HMGB1, high mobility group box 1; PELD, pediatric end-stage liver disease; INR, international normalized ratio; WBC, white blood cell; RBC, red blood cell

### The relationship between intraoperative HMGB1 levels and postoperative PARDS

**Fig. 2:**
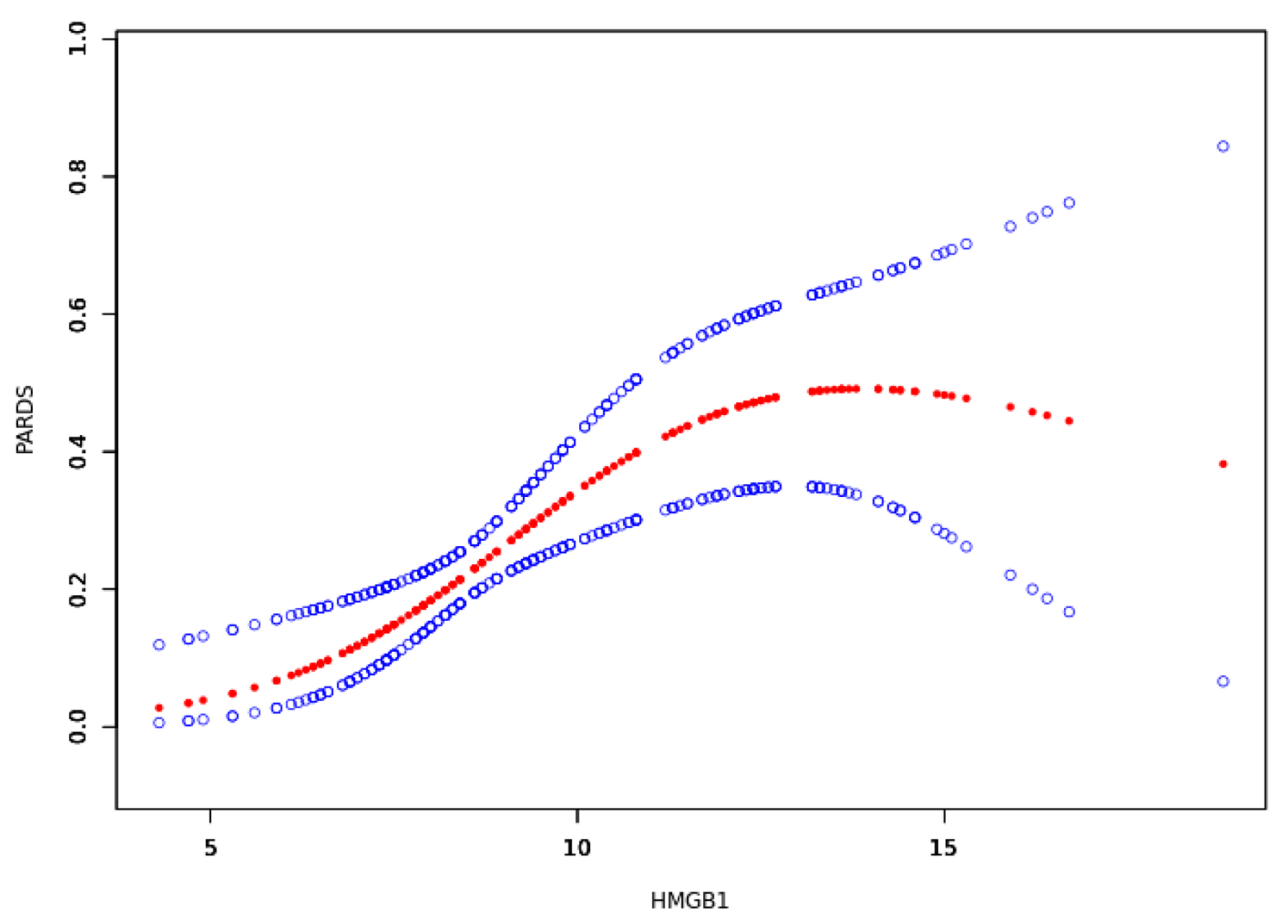
Association between intraoperative serum high mobility group box 1 levels at neohepatic phase 30 min (pg/mL) and the presence of pediatric acute respiratory distress syndrome. The red and blue lines represent the estimated probability and 95% confidence intervals, respectively, for the presence of pediatric acute respiratory distress syndrome and were drawn using the generalized additive model. The model was adjusted for weight (kg), age (months), graft cold ischemia time (min), intraoperative blood loss volume (mL), pretransplant albumin (g/L), and pretransplant total bilirubin (µmol/L)

Multivariate logistic regression analysis was performed following the univariate analysis to further evaluate and confirm the independent effects of serum HMGB1 levels after 30 min reperfusion and postoperative PARDS by using the nonadjusted, adjusted I (age, weight), and adjusted II (age, weight, sex, intraoperative blood loss volume, pretransplant albumin, total bilirubin, and graft cold ischemia time) models. The effect sizes, odds ratios (ORs), and 95% confidence intervals (CIs) are presented in Table 5. PARDS showed a positive association with intraoperative HNGB1 levels (OR 1.28, 95% CI 1.10–1.49, *P* = 0.0017) in the full adjustment model. Furthermore, a spine curve was drawn to visualize the association by a generalized additive model (Fig. 2). It displayed a linear relationship between HMGB1 levels and the probability of early postoperative PARDS.

**Table 5:** Multivariate logistic regression analysis of intraoperative serum high mobility group box 1 levels in the presence of pediatric acute respiratory distress syndrome

Logistic regression model	PARDS		
	Non-adjusted	Adjust I	Adjust II
	OR (95%CI), *P*	OR (95%CI), *P*	OR (95%CI), *P*
HMGB1(pg/ml)	1.41 (1.24, 1.61), < 0.0001	1.40 (1.23, 1.60), < 0.0001	1.28(1.10,1.49), 0.0017
HMGB1(pg/ml)(tertile)			
1st tertile(4.3–8.1 pg/mL)	1.0	1.0	1.0
2nd tertile(8.2-10.6 pg/mL)	5.82 (1.86, 18.27), 0.0025	5.66 (1.79, 17.83), 0.0031	5.25(1.63,16.86), 0.0054
3rd tertile(10.6–18.8 pg/mL)	14.33 (4.71, 43.56), < 0.0001	13.54 (4.42, 41.46), < 0.0001	7.75(2.35,25.57), 0.0008

Adjust I model adjusted for age (month) and weight (kg); adjust II model adjusted for age (month), weight (kg), graft cold ischemia time (min), intraoperative blood loss volume (mL), pretransplant albumin (g/L), and pretransplant total bilirubin (µmol/L)

Abbreviations: PARDS, pediatric acute respiratory distress syndrome; HMGB1, high mobility group box 1; OR, odds ratio; CI, confidence interval

### The results of the subgroup analyses

On the basis of the univariate analysis results, we performed subgroup analyses to further confirm the effect size of intraoperative HMGB1 levels and postoperative PARDS in prespecified and exploratory subgroups (Table 6). Only graft cold ischemia time and weight in tertiles subgroups produced significant differences in different subgroups (*P* for interaction ≤ 0.05). Likewise, no evidence was found of differences in the association of intraoperative HMGB1 levels and postoperative PARDS in the other subgroups analyzed, such as sex, age, intraoperative blood loss volume, pretransplant albumin, and total bilirubin.

**Table 6:** Effect size of intraoperative serum high mobility group box 1 (continuous variable) levels on pediatric acute respiratory distress syndrome in subgroups

Characteristic	No. of participants	PARDS		
		OR(95% CI)	*P*	*P* for interaction
Graft cold ischemia time(min)				0.0118
40–75	68	1.05 (0.78, 1.40)	0.7495	
76–98	71	2.20 (1.33, 3.05)	0.0009	
99–366	71	1.14 (0.90, 1.44)	0.2701	
Intraoperative blood loss volume(mL)				0.4138
50–250	60	1.34 (0.93, 1.93)	0.1160	
300–350	61	1.59 (1.11, 2.28)	0.0113	
400–1000	89	1.22 (1.02, 1.46)	0.0256	
Pretransplant albumin(g/L)				0.8655
23.7–32.6	68	1.30 (1.04, 1.61)	0.0199	
32.7–36.3	72	1.31 (1.01, 1.70)	0.0412	
36.4–49.4	70	1.18 (0.83, 1.66)	0.3536	
Pretransplant total bilirubin(µmol/L)				0.1577
3.15–184.8	70	1.18 (0.87, 1.62)	0.2877	
187.23–326	70	1.19 (0.88, 1.60)	0.2642	
327.5–862.7	70	1.67 (1.25, 2.24)	0.0006	
Weight				0.0084
4.5–6.47	70	1.46 (1.10, 1.93)	0.0080	
6.48–7.46	65	1.96 (1.27, 3.02)	0.0023	
7.5–14.5	75	0.99 (0.77, 1.27)	0.9505	
Sex				0.1544
Male	94	1.17 (0.97, 1.43)	0.1052	
Female	116	1.44 (1.14, 1.81)	0.0019	
Age(month)				0.0835
4.6–6.27	70	1.25 (0.97, 1.60)	0.0837	
6.33–8.8	70	1.68 (1.21, 2.32)	0.0020	
8.9–36.23	70	1.04 (0.77, 1.40)	0.8068	

Logistic regression was performed for subgroup analysis. Age (month), weight (kg), graft cold ischemia time (min), intraoperative blood loss volume (mL), pretransplant albumin (g/L), and pretransplant total bilirubin (µmol/L) were adjusted

Abbreviations: PARDS, pediatric acute respiratory distress syndrome; OR, odds ratio; CI, confidence interval

## Discussion

Pediatric liver transplantation can result in early postoperative complications such as allograft dysfunction and postoperative PARDS, leading to morbidity and mortality. HMGB1 has been identified as an early mediator of inflammation and cellular injury in liver ischemia-reperfusion injury during human orthotopic liver transplantation [21]. To the best of our knowledge, no previous studies have investigated the association between intraoperative serum HMGB1 levels and postoperative PARDS after LDLT in children with biliary atresia. Our Chinese study indicated that serum HMGB1 levels 30 min after reperfusion were positively associated with LDLT-induced PARDS in children with biliary atresia.

According to serum HMGB1 levels at 30 min in the neohepatic phase (Table 2), patients were classified into three tertiles. The pretransplant total bilirubin, graft cold ischemia time, intraoperative blood loss volume, fresh plasma transfusions, RBC transfusions, and surgery time were different among the three groups (*P* ≤ 0.05). In terms of postoperative complications and outcomes, postoperative PARDS increased with the increase of intraoperative HMGB1 levels (*P* < 0.001). Moreover, the postoperative ICU stay time and postoperative respiratory support time were different in the three groups (*P* < 0.001). Previous studies have shown that serum HMGB1 level is positively associated with the severity of liver disease [22], readmission, and mortality [23, 24]. Likewise, the peak concentration of serum HMGB1 is correlated with the length of ICU stay and duration of mechanical ventilation [25]. In one study, a porcine model of cold ischemic liver graft injury was developed; in this model, IL-18 and HMGB1 were found to be early and sensitive indicators of cell damage after prolonged cold ischemic storage [26]. Intraoperative RBC transfusion enhances susceptibility to lung inflammation through the release of HMGB1 and induces necroptosis of lung endothelial cells [27, 28]. Furthermore, Zettel et al. [29]. demonstrated that clearance of *Escherichia coli* by macrophages was inhibited both in vitro and in vivo using supernatant from human-packed RBCs applied in a murine model of trauma and hemorrhagic shock. These results are consistent with those from our study. By contrast, other research has indicated that HMGB1 is not significantly associated with alcoholic hepatitis [30] and transfusion-related acute lung injury [31]; thus, more studies should focus on this area in the future.

As shown in the nonadjusted logistic regression model, HMGB1 was directly correlated with PARDS (OR 1.41, 95% CI 1.24–1.61, *P* < 0.0001). In addition, intraoperative HMGB1 levels at neohepatic phase alternation, pretransplant total bilirubin (*P* < 0.001), intraoperative blood loss volume (*P* = 0.001), and graft cold ischemia time (*P* = 0.047) were associated with the early phase of postoperative PARDS. To make our results more reliable, we conducted sensitivity analyses. The above confounding factors with the addition of age, weight, and preoperative albumin were adjusted respectively in multivariate logistic regression analysis and three models were established. In the final, fully adjusted model, the positive relationship between HMGB1 and PARDS remained unchanged (OR 1.28, 95% CI 1.10–1.49, *P* = 0.0017). This finding means that these confounding factors do not affect the positive association between intraoperative HMGB1 and early PARDS. In addition, we performed subgroup and interaction analyses after adjusting for covariates at different levels in the same model. Finally, we confirmed the linear relationship between HMGB1 and PARDS after drawing a spline curve using a generalized additive model.

ARDS is induced by dysregulated inflammatory response caused by excessive levels of circulating cytokines, which is named as cytokine storm syndrome. Notably, HMGB1 is an important mediator of cytokine storm syndrome. Other studies have demonstrated that HMGB1 is released into the circulation system after hepatic ischemia-reperfusion injury and is related to ARDS. In an animal hepatic ischemia-reperfusion injury model, serum HMGB1 levels were significantly increased immediately after reperfusion. When an adsorption column was used to remove the excessive HMGB1 in serum, liver and lung injuries were reduced [32]. One study showed that HMGB1 levels during liver transplantation were not associated with the development of postoperative ARDS in adults but not children [33]. Moreover, in this study, the confounding factors were not adjusted during the perioperative period. Pediatric patients are more susceptible to surgical stress, ischemia-reperfusion, and the effects of anesthesia drugs and long-term mechanical ventilation because of the specific pediatric immunity state and anatomical features of the respiratory system [17]. As a result, children are more vulnerable to perioperative lung injury than adults. Although studies have suggested that pretransplant PELD scores are associated with postoperative outcome indicators after liver transplantation [12, 34], it was not observed in our study. This may be partly due to differences in study populations, clinical trial conditions, and surgical techniques.

In a recent study [35], HMGB1 released by cholangiocytes was involved in the pathogenesis of biliary atresia and correlated with an increase in afflicted children. We tested the level of serum HMGB1 before transplantation and found no evidence that it was related to postoperative PARDS. This finding suggested that HMGB1, which is related to postoperative PARDS, is released during intraoperative liver ischemia-reperfusion. HMGB1 and nucleosomes could be early mediators of excessive immune activation in liver transplantation and indicate the risk of multiple organ failure and death [36]. HMGB1 in the circulating blood has been shown to cause both experimental and clinical acute lung injury [14]. Plasma levels of HMGB1 are high in patients diagnosed with severe COVID-19, and a significant inverse association exists between serum levels of HMGB1 and clinical outcomes [37]. HMGB1 is exclusively responsible for HMGB1/TLR4/TLR9/NF-κB signaling or TLR4-dependent ROS production and downstream CaMK-mediated signaling [38], which results in further proinflammatory cytokine secretion. Disulfide-HMGB1 secreted can then potentially mediate a positive feedback loop by binding to TLR4 and initiating a downstream pro-inflammatory signaling cascade, which can further induce cell death. In addition, HMGB1 disrupts the integrity of the endothelial barrier through the advanced glycation end products/Rho-associated kinase 1 pathway, which induces stress fiber formation in the short term via phosphorylation of the myosin light chain [39].

In our study, the incidence of PARDS after 7 days of transplantation was 26.19%, which is higher than that in previous reports [19, 33]. This difference was partly because we used the specific definition of PARDS recommended at the Pediatric Acute Lung Injury Consensus Conference and developed by an Expert Consensus Meeting in 2015 [10]. The symptoms of hypoxemia and radiographic changes must occur within 7 days of a known clinical insult to qualify for PARDS. The definition of ARDS was created in Berlin in 2012 [40], and this definition focuses on adult lung injury and has limitations when applied to children. The noninvasive measurement of SpO_2_ was added to the definition of PARDS in the 2015 definition. Consequently, the Berlin definition that requires the direct measurement of PaO_2_ could underestimate the prevalence of ARDS in children. In addition, some pediatric practitioners use the oxygenation index and OSI instead of the PaO_2_/FiO_2_ ratio. The oxygenation index and OSI are greatly influenced by ventilator pressure [41, 42], particularly in the pediatric ICU where there is greater variability in ventilator management relative to the adult ICU [43, 44].

Our study is the first to find that elevated levels of HMGB1 in the neohepatic phase are associated with PARDS that occurs within 7 days of LDLT, regardless of age, weight, pretransplant albumin, total bilirubin, graft cold ischemia time, and intraoperative blood loss volume. Although the treatment strategy to prevent intraoperative ischemia-reperfusion injury is not well understood, understanding this parameter may be beneficial in children at potential risk of PARDS after liver transplantation. Therefore, our study findings suggest that if abnormal levels of HMGB1 levels are detected intraoperatively, physicians need to consider the possibility of early PARDS developing postoperatively. Accordingly, preventive measures should be considered in advance, and lung protective strategies and fluid management should be included during the perioperative period. Furthermore, the child’s condition should be closely monitored after surgery to ensure early detection and early treatment to prevent serious respiratory tract damage.

Predictive models of PARDS after LDLT have long been a hot topic. However, the prerequisite for building a reliable model is to understand the relationship between each predictor and PARDS after LDLT, and the nonlinear relationship is very important. In this study, we found that the relationship between intraoperative and postoperative PARDS is nonlinear. This knowledge will improve clinical models in the future.

There are several limitations to our study. Firstly, the retrospective study design and outcome reliability are dependent on the accuracy and completeness of medical records and surgical reports. Consequently, a larger sample size could be used in the future to examine the effects of individual variables. Secondly, only the total concentration of HMGB1 in plasma was analyzed, and subtypes were not classified. Partially oxidized disulfide-HMGB1 redox form is a more relevant mechanistic biomarker and therapeutic target than total HMGB1 in acute lung injury induced by liver transplantation [21]. Moreover, we could not determine dynamic changes in serum HMGB1 levels during the whole perioperative period because of the limitation of the raw data. Thirdly, only children in China were included; therefore, the results might not be applicable to adults or individuals of other ethnic groups. In addition, patients with perinatal cardiopulmonary disease and acute respiratory infection were excluded, so studies in such populations should be conducted. Finally, our study focused on HMGB1 as an inflammatory mediator, whereas there are many inflammatory cytokines, such as tumor necrosis factor-α, interleukin-6, interleukin-8, and endotoxin, for which data are not available. Therefore, additional clinical studies are needed to focus on the overall association changes of clinical prognosis in the future.

The main strength of this study is that it provides important information for the prevention of PARDS after LDLT in children with biliary atresia. Compared with other studies of lung injury following liver transplantation, our study was conducted in children with biliary atresia who had undergone LDLT, and dynamic changes in serum HMGB1 levels may reflect minor alterations in distal organ inflammatory responses. Our findings suggest that targeting acute inflammation could potentially alleviate early systemic inflammatory response syndrome a graft dysfunction, and alleviates or prevents long-term complications.

## Conclusion

In conclusion, there is a positive relationship between serum HMGB1 levels 30 min after reperfusion and PARDS during the first week after LDLT in children with biliary atresia in China.

## Electronic supplementary material

Below is the link to the electronic supplementary material.

Extended Data Table 1: Effect size of intraoperative serum high mobility group box 1 (continuous variable) levels on pediatric acute respiratory distress syndrome in pretransplant PELD score subgroups

## Data Availability

The datasets used and/or analyzed during the current study are available from the corresponding author upon reasonable request.

## Abbreviations

HMGB1: High mobility group box 1
LDTL: Living donor liver transplantation
PARDS: Pediatric acute respiratory distress syndrome
ARDS: Acute respiratory distress syndrome
PELD: Pediatric end-stage liver disease
RBCs: Red blood cells
OR: Odds ratio
95% CI: 95% Confidence interval
OI: Oxygenation index
OSI: Oxygen saturation index
