# Supplementary material for: Association between serum HMGB1 elevation and early pediatric acute respiratory distress syndrome: a retrospective study of pediatric living donor liver transplant recipients with biliary atresia in China
Source: BMC Anesthesiol. 2023 Mar 21;23:87. doi: 10.1186/s12871-023-02040-0 (PMC10028322; doi:10.1186/s12871-023-02040-0)
Supplement: Supplementary file 1 — Extended Data Table 1: Effect size of intraoperative serum high mobility group box 1 (continuous variable) levels on pediatric acute respiratory distress syndrome in pretransplant PELD score subgroups [file 12871_2023_2040_MOESM1_ESM.docx]

**Extended Data Table 1** Effect size of intraoperative serum high mobility group box 1 (continuous variable) levels on pediatric acute respiratory distress syndrome in pretransplant PELD score subgroups

| Characteristic | No. of participants | PARDS |  |
| --- | --- | --- | --- |
|  |  | OR(95% CI) | *P* |
| Pretransplant PELD score |  |  |  |
| 0-13 | 69 | 1.17(0.88, 1.57) | 0.2798 |
| 14-22 | 72 | 1.17(0.88, 1.54) | 0.2781 |
| 23-51 | 69 | 1.45(1.07, 1.96) | 0.0156 |

Logistic regression was performed for subgroup analysis. Age (month), weight (kg), graft cold ischemia time (min), intraoperative blood loss volume (mL), pretransplant albumin (g/L), and pretransplant total bilirubin (μmol/L) were adjusted.

Abbreviations: PARDS, pediatric acute respiratory distress syndrome; OR, odds ratio; CI, confidence interval.
